# Clinical Imaging-Derived Metrics of Corticospinal Tract Structural Integrity Are Associated With Post-stroke Motor Outcomes: A Retrospective Study

**DOI:** 10.3389/fneur.2022.804133

**Published:** 2022-02-17

**Authors:** Mary Alice Saltão da Silva, Nathan Allen Baune, Samir Belagaje, Michael R. Borich

**Affiliations:** ^1^Neural Plasticity Research Laboratory, Division of Physical Therapy, Department of Rehabilitation Medicine, Emory University School of Medicine, Atlanta, GA, United States; ^2^School of Biological Sciences, Georgia Institute of Technology, Atlanta, GA, United States; ^3^Departments of Neurology and Rehabilitation Medicine, Emory University School of Medicine, Atlanta, GA, United States

**Keywords:** stroke, clinical MRI, motor recovery, outcome prediction, premotor and motor cortex, corticospinal tract (CST)

## Abstract

**Objective:**

The primary objective of this study was to retrospectively investigate associations between clinical magnetic resonance imaging-based (MRI) metrics of corticospinal tract (CST) status and paretic upper extremity (PUE) motor recovery in patients that completed acute inpatient rehabilitation (AR) post-stroke.

**Methods:**

We conducted a longitudinal chart review of patients post-stroke who received care in the Emory University Hospital system during acute hospitalization, AR, and outpatient therapy. We extracted demographic information, stroke characteristics, and longitudinal documentation of post-stroke motor function from institutional electronic medical records. Serial assessments of paretic shoulder abduction and finger extension were estimated (E-SAFE) and an estimated Action Research Arm Test (E-ARAT) score was used to quantify 3-month PUE motor function outcome. Clinically-diagnostic MRI were used to create lesion masks that were spatially normalized and overlaid onto a white matter tract atlas delineating CST contributions emanating from six cortical seed regions to obtain the percentage of CST lesion overlap. Metric associations were investigated with correlation and cluster analyses, Kruskal-Wallis tests, classification and regression tree analysis.

**Results:**

Thirty-four patients met study eligibility criteria. All CST overlap percentages were correlated with E-ARAT however, ventral premotor tract (PMv) overlap was the only tract that remained significantly correlated after multiple comparisons adjustment. Lesion overlap percentage in CST contributions from all seed regions was significantly different between outcome categories. Using MRI metrics alone, dorsal premotor (PMd) and PMv tracts classified recovery outcome category with 79.4% accuracy. When clinical and MRI metrics were combined, AR E-SAFE, patient age, and overall CST lesion overlap classified patients with 88.2% accuracy.

**Conclusions:**

Study findings revealed clinical MRI-derived CST lesion overlap was associated with PUE motor outcome post-stroke and that cortical projections within the CST, particularly those emanating from non-M1 cortical areas, prominently ventral premotor (PMv) and dorsal premotor (PMd) cortices, distinguished between PUE outcome groups. Exploratory predictive models using clinical MRI metrics, either alone or in combination with clinical measures, were able to accurately identify recovery outcome category for the study cohort during both the acute and early subacute phases of post-stroke recovery. Prospective studies are recommended to determine the predictive utility of including clinical imaging-based biomarkers of white matter tract structural integrity in predictive models of post-stroke recovery.

## Introduction

Stroke is a leading cause of long-term adult disability in the United States (US) (1). Early, accurate prediction of recovery of motor function post-stroke would enable precision-based rehabilitation strategies to improve outcomes and reduce disability (2, 3). However, current clinical practice lacks validated objective tools necessary to accurately predict motor recovery and deliver optimally-targeted interventions (1, 2, 4, 5).

The majority of motor recovery occurs early after stroke, typically plateauing around 3-months post-injury, and is thought to be primarily regulated by molecular mechanisms underlying structural and functional reorganization of the motor system within both lesioned and non-lesioned hemispheres (6–9). The corticospinal tract (CST) is the canonical descending motor output pathway responsible for generating voluntary movements and is particularly important for fine motor control of dexterous distal movements in both animals and humans (6, 9, 10). Approximately 40% of the CST originates directly from the primary motor cortex (M1), while an additional ~30% of the CST is comprised of tracts originating from non-M1 motor areas such as premotor cortex (PM), supplementary motor cortex (SMA), and cingulate motor areas (11–14). Pyramidal cells within M1 generate signals for execution of movements in context (15). PM and cingulate regions are known to be involved in control of both the cognitive aspects of motor planning (including spatial attention) and the execution of movement itself, while the SMA is thought to contribute to temporal specificity of muscle activation, particularly during reaching movements (11, 16–20). The CST also encompasses projections from the primary somatosensory cortex (S1) to the SC suppling sensory information that informs movement output, enabling precision and refinement of motor control (21). Stroke-induced disruption of the CST often results in functional impairment of the hand and upper limb and is known to particularly affect the recovery of fine motor control (7, 22, 23). Prior studies have shown associations between paretic upper extremity (PUE) motor recovery and disruptions of M1 contributions to the CST post-stroke (22–25). More recently, other studies have evaluated differential contributors to CST structural integrity with inconclusive results (16, 25–29). Thus, less is currently known about the relevance of non-M1 projections within the CST to specific elements of PUE motor recovery.

Models to predict PUE motor recovery outcome have been developed and implemented in other healthcare systems (30–35). The Predict Recovery Potential (PREP2) prediction tool, developed and internally validated in New Zealand, predicts PUE motor outcomes using a combination of clinical assessments and objective neurological biomarkers (30). PREP2 employs transcranial magnetic stimulation (TMS) of M1 to measure CST functional integrity and the National Institutes of Health Stroke Scale (NIHSS) score to differentiate functional prognosis in the subset of individuals with initially low PUE strength (30, 36). TMS assessment is not currently standard-of-care in US hospitals however, clinical neuroimaging is routinely used to diagnose stroke in the US and can be used to quantify structural integrity of the CST.

Using magnetic resonance imaging (MRI), the structural integrity of the descending sensorimotor system can be quantified by measuring both the location and extent of stroke lesion overlap with the CST and has been used to identify how damage to anatomic structures relates to post-stroke motor outcomes (25–27, 32, 37–39). Several studies have shown that poorer motor outcomes are correlated with a greater extent of lesion encroachment within the CST (22–25, 39). Interestingly, structural MRI may outperform the use of clinical bedside measures of PUE strength or functional impairment (3, 23, 32, 40), but most of these studies employed research-grade MRI with higher resolution compared to standard-of-care clinical MRI (27). In the absence of both TMS and research-grade MRI, routine acute clinical MRI may offer alternative estimates of lesion overlap and anatomical integrity that are already available. In fact, studies using clinical MRI have emerged providing high quality evidence for imaging-derived prediction of motor return post-stroke, but have yet to combine those standard-of-care, diagnostic MRI metrics with clinical measures to predict functional outcome (3, 22, 26, 27, 41).

Previously, we observed that estimated shoulder abduction and finger extension (E-SAFE) PUE strength from assessments at admission to acute inpatient rehabilitation (AR) could distinguish PUE motor recovery outcomes with 70% accuracy but that clinical metrics alone were unable to distinguish between *Limited* and *Poor* recovery outcome groups (42). Further, most previous work has not evaluated MRI prognostic utility for hemorrhagic stroke (22, 27, 41). Accordingly, there is a need to investigate possible markers of CST integrity that differentiate outcomes for both ischemic and intracerebral hemorrhagic strokes and is of particular importance for those patients with initially-lower levels of volitional control who exhibit the most difficult to predict recovery patterns (27, 30, 43, 44).

The primary objective of this study was to retrospectively investigate associations between clinical MRI-based metrics of CST status and PUE motor recovery in patients that completed AR post-stroke. We predicted that clinical MRI-based measures of lesion disruption to M1 and non-M1 contributions to the CST would be associated with PUE function outcome at ~90 days post-stroke. Our exploratory prediction was that metrics of lesion-based CST disruption would improve the predictive accuracy of PUE motor recovery outcome over use of clinical metrics alone, particularly for those with initially lower levels of PUE strength.

## Materials and Methods

### Study Population and Selection Criteria

We conducted a longitudinal retrospective chart review of all patients admitted with a primary diagnosis of stroke to Emory University Hospital (EUH), a representative, urban, academic, comprehensive stroke care center in the US, between September 1, 2016 and August 31, 2018. Using previously established inclusion and exclusion criteria, we identified eligible patients (30). Major inclusion criteria included the following: first ever or recurrent, ischemic or intracerebral hemorrhagic stroke; new upper extremity weakness beginning at or after current stroke onset; over the age of 18 years (30). In addition, individuals were required to have remained within the EUH system for acute hospitalization, acute inpatient rehabilitation at Emory Rehabilitation Hospital, and Emory outpatient therapy through at least 90 days post-stroke to permit longitudinal assessment of PUE recovery outcomes and reduce the heterogeneity of post-stroke care for the study cohort across the continuum of recovery. Lastly, patients were required to have received clinically-diagnostic MRI during their acute stroke workup at EUH. This study received Emory University Institutional Review Board approval and patient consent was waived.

### Data Extraction and Analysis

#### Clinical Variables

As previously described, clinical metrics including demographic information, stroke characteristics, care continuum metrics, and provider documentation of post-stoke motor function were extracted from Cerner Powerchart, the institutional electronic medical record system of the Emory Healthcare system (42).

Provider documentation of PUE strength and post-stroke disability included manual muscle test scores, sensation, coordination, language impairments, and measures of mobility. These metrics were recorded serially by different providers within the care continuum including physicians, physical therapists, occupational therapists, and speech language pathologists [data extraction methodology detailed in (42)]. Shoulder abduction (SA) and finger extension (FE) manual muscle tests were used to calculate a SAFE score (/10) for each patient (30, 32, 45). If an objective SAFE score was not available in clinical documentation, an E-SAFE score was calculated using available assessments of PUE strength with preference given to strength of muscles with similar spinal cord segmental innervation (46, 47). If the E-SAFE score was documented more than once during acute hospitalization, the assessment performed closest to inpatient day-3 was used; in the AR setting, the E-SAFE score performed closest to admission was used, in accordance with previous work (30, 32, 38, 42).

The Action Research Arm Test (ARAT) was used as the primary dependent variable to quantify PUE functional outcome for each patient. The ARAT is a validated, sensitive, and reliable test, commonly used in stroke-related research to measure level of upper extremity function (48). Due to the retrospective nature of the study design, ARAT scores were estimated from therapy documentation at ~90 days post-stroke in accordance with the grading criteria for each test. Estimated ARAT (E-ARAT) scoring was conducted by two licensed, clinical neurologic therapists who were otherwise blinded to study findings. Rehabilitation provider notes were evaluated in detail to extract the following measures for each patient: clinical assessments of PUE muscle and grip strength, coordination, active and passive range of motion, observational movement analysis, therapeutic activity, exercises performed, rehabilitation goals, Nine-Hole Peg Test and Box and Block Test scores as compared to matched, normative values (49–52). Each clinician independently reviewed the electronic medical record and determined maximal and minimal scores for each ARAT test item, creating a score range for every patient. E-ARAT for every patient was calculated by taking the median score from both clinicians and averaging the two values.

Previously reported three-cluster cluster analysis produced distinct outcome groups with centers at least 12 points apart (the minimal clinically important difference) on the E-ARAT and were defined as *Good, Limited*, and *Poor* PUE outcome groups, corresponding to diminishing levels of PUE function (42).

#### Image Processing and Lesion Mapping

Standard-of-care clinical MRI were obtained from the Department of Radiology at EUH. Stroke topography was determined using diagnostic, clinically-obtained T2-weighted images. Diffusion weighted images were utilized for ischemic strokes and gradient echo images were used for hemorrhagic strokes in order to maximize visual contrast and improve the specificity of lesion identification. Scans performed closest to the date of admission were used when multiple MRI sequences were acquired during the acute inpatient stay. Lesion masks were created in ITK-SNAP version 3.8.0 (53) by a member of the research staff who was otherwise blinded to participant outcomes. Lesions were traced in a slice-by-slice manner in the axial plane using a semi-automated segmentation process. In this process, a scalar “speed” image was created to delineate between structures of interest (53, 54). Active contour segmentation was then guided by both the speed image and manually-placed initialization seeds (54). Traces were manually adjusted as necessary in the sagittal and coronal planes to ensure accuracy of the three-dimensional segmentation. Once drawn, lesion mask location and extent were independently verified visually and with neuroradiology documentation. A board-certified vascular neurologist (S.B.) provided additional consultation to ensure accuracy of lesion masks. Lesion volume was automatically calculated by ITK-SNAP software (53).

T1-weighted images (anatomical scans), T2-weighted images (pathological scans), and lesion masks (lesion map) were used as inputs for spatial normalization into standard Montreal Neurological Institute (MNI) space using Statistical Parametric Mapping software (SPM12) (55, 56). SPM's combined normalization-segmentation process was employed via the associated clinical toolbox using the 2 mm T1-weighted MNI152 template, a standard template bounding box [−90 −126 −72; 90 90 108], and 2 mm^3^ voxel size (55–57). Validation of normalization in standard stereotaxic space was then visually confirmed to ensure proper alignment of cortical boundaries, subcortical anatomical landmarks, and drawn lesions.

#### CST Lesion Overlap Calculation

The spatially normalized lesion mask for each participant was processed through custom MNI ROI overlap software to obtain CST lesion overlap using the sensorimotor area tract template (SMATT) atlas (58, 59). The SMATT atlas delineates contributions to the CST emanating from six cortical seed regions: M1; ventral and dorsal premotor areas (PMv and PMd); supplementary and pre-supplementary motor areas (SMA and preSMA); and primary somatosensory cortex (S1) (58). SMATT was created using a slice-by-slice thresholding technique in both right and left hemispheres to minimize tract overlap while conserving tract volume (58). Data analysis output included voxel sizes for each tract, the number of voxels disrupted by the lesion, and percent tract lesion overlap. The lesion load output was individuated by seed region (M1, PMv, PMd, SMA, preSMA, and S1), therefore a whole CST lesion overlap percentage (CST overlap) was calculated by summing the number of voxels in each tract, the number of voxels overlapped by the region and dividing the two metrics. This calculation was conducted using tract voxel numbers for the affected hemisphere, as there are slight differences in CST size between right and left hemispheres (58). A non-M1 CST lesion overlap percentage was calculated using similar methodology, but omitting overlap data from the M1 CST only. CST lesion overlap percentage was also calculated using the Johns Hopkins University white matter tractography atlas (JHU) (60). The JHU atlas has been employed more often in tractography studies, so it was used to comparatively assess SMATT atlas utility (27, 60).

#### Statistical Methodology

Descriptive analysis was performed to summarize the distribution of variables of interest for the entire cohort. Non-parametric correlation analyses (Spearman's rho, r_S_) were performed to evaluate the relationship between CST lesion overlap metrics, lesion volume, and level of paretic upper extremity motor function at 3-months post-stroke (E-ARAT scores). Parametric correlation analyses (Pearson's correlation coefficient, r) were performed to evaluate the relationship between continuous MRI variables. Independent-samples means comparisons were then conducted using Kruskal-Wallis tests to identify differences in MRI metrics between outcome groups and to evaluate the effects of stroke type on PUE motor function outcome. To explore which MRI-derived factor(s) may predict outcome cluster group, a classification and regression tree (CART) analysis was conducted. Gini was used to maximize homogeneity of child nodes with respect to the value of the target variable. Clinical and MRI metrics including all tract overlap percentages from both SMATT and JHU atlases, lesion volume, stroke characteristics, patient age, patient comorbidities, E-SAFE scores, sensation, coordination, language impairments, and measures of mobility were available as inputs using a maximum tree depth of 2, a minimum terminal node size of 3, and automated pruning to avoid over-fitting. Positive (PPV) and negative (NPV) predictive values, sensitivity, and specificity of the resulting decision tree were also calculated. The interrater reliability of the E-ARAT scores conducted by the two clinician raters was assessed with an intraclass correlation coefficient (ICC), calculated using a two-way mixed effects model, considering people effects to be random and item effects to be fixed (48, 61).

Tests were two-tailed with significance set to *p* < 0.05. Significance values were adjusted for multiple comparisons using Bonferroni correction with a two-tailed significance level of *p* = 0.0083 for correlation analyses (0.05/6 comparisons and *p* = 0.02 for *t*-tests) (0.05/3 comparisons). All statistical analyses were conducted using IBM® Statistical Package for the Social Sciences (SPSS).

## Results

**Figure 1:**
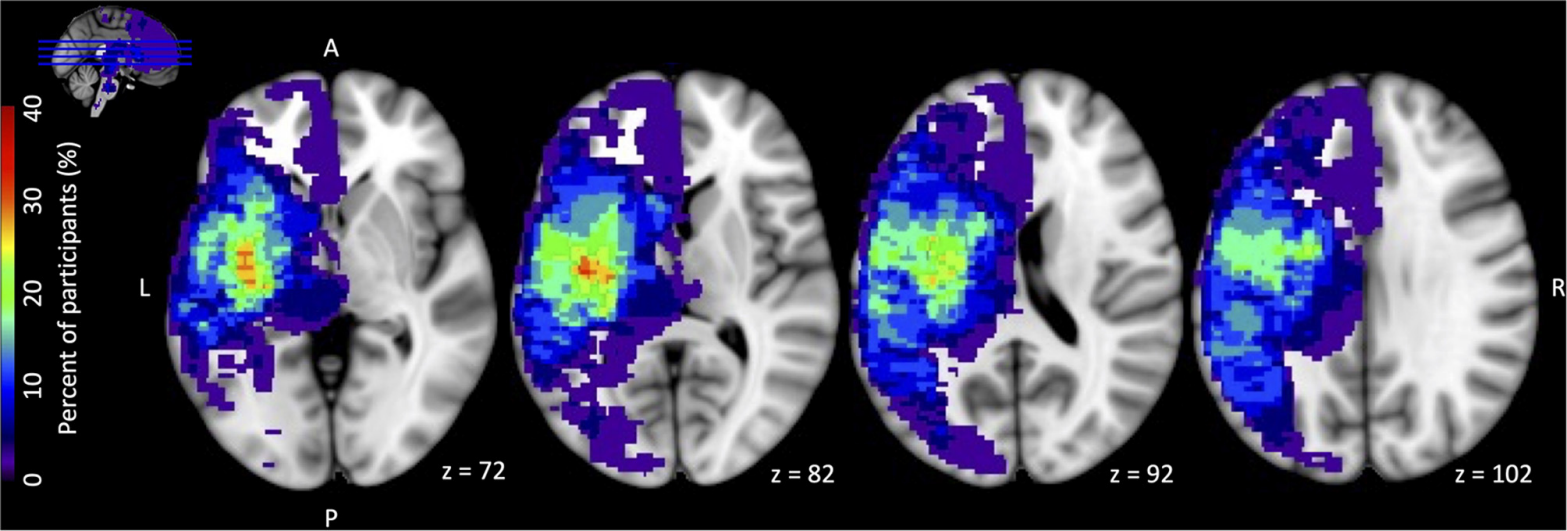
Stroke lesion overlap heat map for all 34 participants. All lesions were flipped onto the left hemisphere for display. For the 5 participants with stroke involvement in bilateral hemispheres, the hemisphere contralateral to the affected paretic upper extremity was used for stroke location purposes. Color bar on the left has a maximum value 40% = 13 participants (maximal overlap voxel = red).

Of the 599 patients admitted to EUH with a primary diagnosis of stroke during fiscal years 2016–2018, 34 patients [median age: 64 (36–84) years, female: 14] met full study eligibility criteria. Twenty-five patients were diagnosed with ischemic stroke (70.6%), 8 with hemorrhagic stroke (23.5%), and 2 with ischemic stroke with hemorrhagic conversion (5.9%). Twelve strokes (35.3%) were localized in the right hemisphere, 17 (50.0%) in the left hemisphere, and 5 (14.7%) had bilateral involvement. Twenty-nine (85.3% of strokes) had subcortical involvement; 4 (11.8%) were localized to the brainstem. Seven patients (20.6%) had previous clinical stroke while 24 (70.6%) had some degree of white matter disease. A lesion heat map for all 34 participants is depicted in Figure 1. The median time to Acute E-SAFE assessment was 3.0 days (range = 0–12 days) and required estimation for 91% of patients. The median time to AR SAFE evaluation was 7 days (range = 2–27 days) and required estimation in 97% of patients. The median time to MRI was 1 day (range = 0–6 days). The median time to E-ARAT assessment was 90.5 days (range = 69–428 days) (Table 1). Interrater agreement for E-ARAT scores was high (ICC = 0.846, 95% CI: 0.69–0.92, *p* < 0.0005). Additional patient characteristics have been summarized previously (42).

**Table 1:** PUE outcome cluster group data.

**PUE recovery outcome group**	**Good**	**Limited**	**Poor**
	**Median (range)**	**Median (range)**	**Median (range)**
Number of individuals (% total)	18 (52.9%)	12 (35.3%)	4 (11.8%)
E-ARAT^a^ score* (/57)	42.3 (35–50.8)	28.13 (18.5–33.5)	11.5 (1.5–14.8)
Acute E-SAFE^b^ score** (/10)	6 (1–8)	3 (0–8)	0 (0–0)
AR^c^ E-SAFE^b^ score*** (/10)	8 (4–10)	3.5 (0–8)	0.5 (0–2)
Acute LOS^d^ (EUH), days	7 (2–25)	6.5 (2–27)	6 (1–23)
AR^c^ LOS^d^ (ERH), days	19 (6–35)	20 (7–35)	19.5 (17–25)
Outpatient therapy duration, days	99.5 (44–314)	82 (37–271)	71 (29–157)
Number of outpatient visits	20.5 (12–50)	23.5 (11–54)	18 (7–22)
Lesion volume (mm^3^)	6,182 (450–169,300)	28,645 (180–153,300)	77,495 (4,705–163,000)
Total CST^e^ load (%) (SMATT)^f^	3.9 (0.0–22.5)	11.5 (6.0–35.1)	31.7 (13.0–61.8)
Number of SMATT^f^ CST^e^ tracts affected	5 (0–6)	6 (3–6)	6 (6–6)

a *E-ARAT, estimated ARAT*.

b *E-SAFE, estimated SAFE*.

c *AR, acute inpatient rehabilitation*.

d *LOS, length of stay*.

e *CST, corticospinal tract*.

f *SMATT, Sensorimotor area tract template*.

* *34/34 scores estimated*;

** *31/34 (91%) scores estimated*;

*** *33/34 (97%) scores estimated*.

### Correlation Analyses

**Figure 2:**
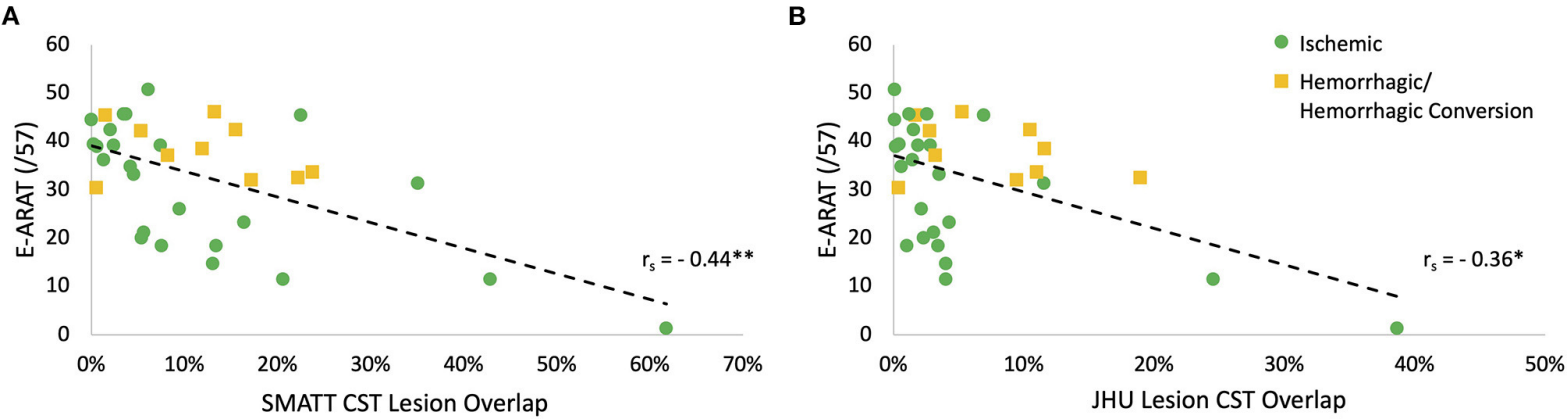
Corticospinal tract (CST) lesion overlap is correlated with paretic upper extremity (PUE) motor outcome. **(A)** Sensorimotor area tract template (SMATT) CST lesion overlap is moderately correlated with estimated Action Research Arm Test (E-ARAT) score; (r_s_ = −0.44, *n* = 34, **p = 0.0087). **(B)** Johns Hopkins University (JHU) atlas CST lesion overlap is weakly correlated with E-ARAT score; (*r*_s_ = −0.36, *n* = 34, *p = 0.036). *Correlation is significant to the 0.05 level (two-tailed); **Correlation is significant to the 0.01 level (two-tailed).

Spearman's correlation analyses revealed the SMATT CST overlap to be moderately negatively correlated with E-ARAT [SMATT CST r_s_ (32) = −0.443, *p* = 0.0087] (Figure 2A). The JHU CST overlap was also significantly correlated with E-ARAT, though less strongly [JHU CST r_s_ (32) = −0.361, *p* = 0.036] (Figure 2B). JHU CST and SMATT CST overlap were highly and significantly correlated [r (32) = 0.919, *p* < 0.0001]. Lesion volume was not associated with E-ARAT scores [lesion volume r_s_ (32) = −0.071, *p* = 0.69].

Further correlation analyses were conducted to evaluate which regions within the SMATT atlas were most highly correlated with the E-ARAT. PMv overlap percentage was the only tract that remained significantly correlated after adjusting for multiple comparisons [r_s_ (32) = −0.457, *p* = 0.0066] (see Table 2).

**Table 2:** SMATT tracts correlate with E-ARAT scores and distinguish between PUE outcome groups (*n* = 34).

**Tract** **name**	**Spearman** **correlations**	**Kruskal-Wallis** **tests**	**Pairwise comparisons** **(median difference in %** ***overlap, p*****^a^)**
SMATT CST	r_s_ (32) = −0.443, *p* = 0.0087*	H (2) = 11.41, *p* = 0.003*	*Good-Poor*: 20.25, p^a^=0.007** Good-Limited: 7.55, *p*^a^ = 0.072^†^
M1	r_s_ (32) = −0.344, *p* = 0.046*	H (2) = 7.84, *p* = 0.02*	*Good*-*Poor*: 25.25, *p*^a^ = 0.024**
PMd	r_s_ (32) = −0.413, *p* = 0.015*	H (2) = 12.15, *p* = 0.002**	*Good*-*Poor*: 32.10, *p*^a^ = 0.002**
			*Limited*-*Poor*: 23.76, *p*^a^ = 0.073^†^
PMv	r_s_ (32) = −0.457, *p* = 0.0066**	H (2) = 13.66, *p* = 0.001**	*Good*-*Poor*: 53.28, *p*^a^ = 0.005**
			*Good*-*Limited*: 10.36, *p*^a^ = 0.018**
preSMA	r_s_ (32) = −0.414, *p* = 0.015*	H (2) = 10.65, *p* = 0.005**	*Good*-*Poor*: 29.08, *p*^a^ = 0.004**
			*Limited*-*Poor*: 22.79, *p*^a^ = 0.084^†^
SMA	r_s_ (32) = −0.375, *p* = 0.029*	H (2) = 11.54, *p* = 0.003**	*Good*-*Poor*: 28.88, *p*^a^ = 0.004**
S1	r_s_ (32) = −0.381, *p* = 0.026*	H (2) = 7.02, *p* = 0.03*	*Good*-*Poor*: 18.79, *p*^a^ = 0.057^†^

* *Correlation is significant*.

** *Correlation remained significant after Bonferroni correction. All p-values reported for pairwise comparisons (last column) represent adjusted significance (p^a^)*.

† *Approaching significance after Bonferroni correction*.

### Kruskal-Wallis Results

**Figure 3:**
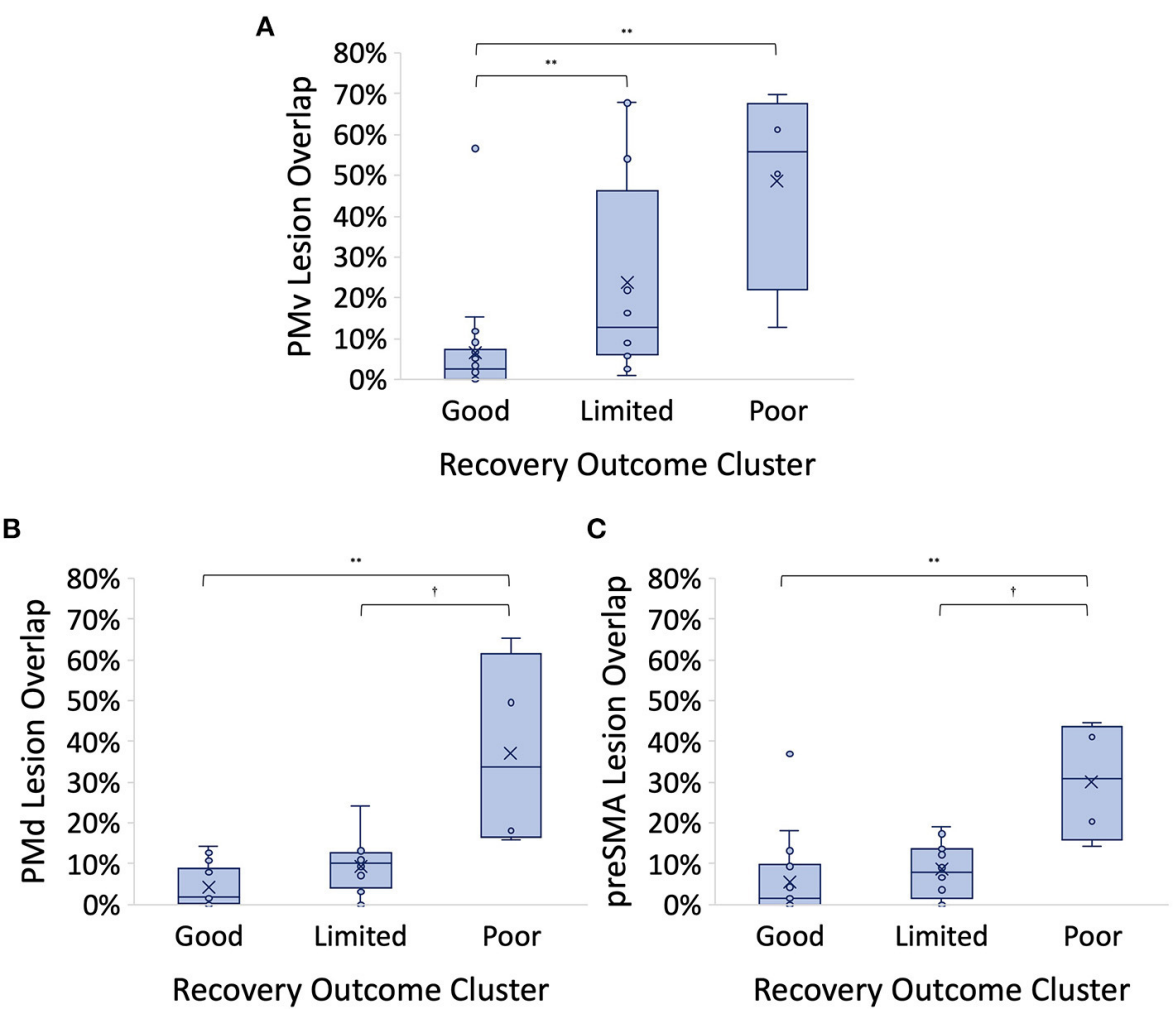
**(A)** Ventral premotor (PMv) corticospinal tract (CST) lesion overlap % is higher for those in the *Good* outcome group over those in both the *Limited and Poor* outcome groups. *Good-Limited p* = 0.018, *Good-Poor p* = 0.005. **(B)** Dorsal premotor (PMd) CST lesion overlap % is higher for those in the *Good* outcome group over those in the *Poor* outcome group and showed a non-significant trend for a difference between *Limited* and *Poor* outcome groups. *Good-Poor p* = 0.002, *Limited-Poor p* = 0.073. **(C)** Pre-supplementary motor (preSMA) CST lesion overlap % is higher for those in the *Good* outcome group over those in the *Poor* outcome group and showed a non-significant trend for a difference between *Limited* and *Poor* outcome groups. *Good-Poor p* = 0.004, *Limited-Poor p* = 0.084. All *p* values reported represent adjusted significance; Cluster centers denoted with “x” in the figure; horizontal bars represent medians *p < 0.05 level, **p < 0.01 level; ^†^Approaching significance after Bonferroni correction.

The Kruskal-Wallis tests showed significant differences between outcome groups for all SMATT tract lesion overlap percentages. *Post-hoc* pairwise comparisons revealed that almost all significant differences were between *Good* and *Poor* outcome groups. Only PMv lesion overlap revealed a significant difference between *Good* and *Limited* outcome groups (*Good-Limited* median difference = 10.36%, *p* = 0.018) (Figure 3A), though SMATT CST lesion overlap showed a non-significant trend for a difference between *Good* and *Limited* outcome groups after Bonferroni correction (SMATT CST *Good-Limited* median difference = 7.55%, *p* = 0.072). See Table 2, pairwise comparisons. No MRI variable significantly differentiated the *Limited* from *Poor* outcome groups, though overlap percentages from 2 non-M1 CST contributors, PMd and preSMA, were both approaching significance after Bonferroni correction (PMd *Limited-Poor p* = 0.073, preSMA *Limited-Poor p* = 0.084) (Figures 3B,C). Lesion volume was not significantly associated with PUE outcome category [H (2) = 2.06, *p* = 0.36]. PUE outcome (E-ARAT) was not significantly different for those with ischemic vs. hemorrhagic stroke [H (1) = 1.46, *p* = 0.23]. Lengths of stay in both acute and rehabilitation hospitals were not significantly different between groups, nor was the duration of outpatient therapy or number of outpatient visits different (Table 1).

**Figure 4:**
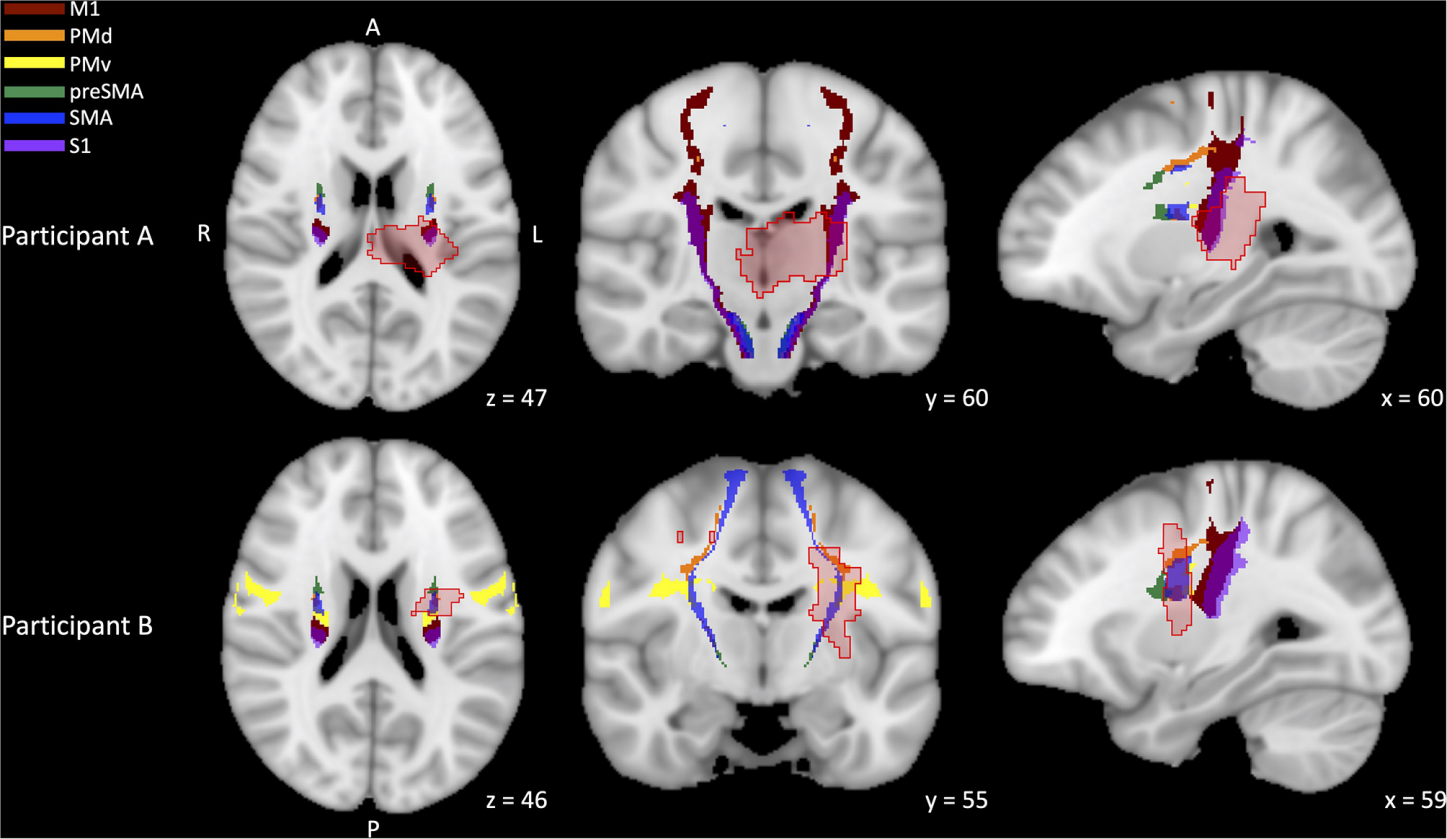
Representative stroke lesions and sensorimotor area tract template (SMATT) corticospinal tract (CST) templates. CST templates have been differentiated by contributing region: primary motor cortex (M1, dark red), dorsal premotor cortex (PMd, orange), ventral premotor cortex (PMv, yellow), pre-supplementary motor cortex (preSMA, green), supplementary motor cortex (SMA, blue), primary somatosensory cortex (S1, purple). Stroke lesions are depicted in light red with dark red outline. Participant A [top (right, middle, left)] achieved a PUE outcome in the *Good* category. Participant B bottom (right, middle, left) achieved a PUE outcome in the *Limited* category. Right, Middle, and Left slices depict the axial, coronal, and sagittal slices, respectively. Both individuals had similar whole CST lesion overlap (A = 9.51%, B = 11.97%) but participant A had higher relative contribution of M1 CST lesion overlap (A M1 overlap = 18.87%, participant B M1 overlap = 1.94%).

Figure 4 depicts representative lesions overlaid on the SMATT atlas template for 2 individuals from different outcome groups. Participant A (Figure 4, top right, middle, left) achieved a PUE outcome in the *Good* category. Participant B (Figure 4, bottom right, middle, left) achieved a PUE outcome in the *Limited* category. Right, Middle, and Left slices depict the axial, coronal, and sagittal slices, respectively. Stroke lesions are depicted in light red with dark red outline. Individual contributions to the CST are color coded (see figure key). Both individuals had similar whole CST lesion overlap (participant A = 9.51%, participant B = 11.97%) but participant A had higher relative contribution of M1 CST lesion overlap (Participant A M1 overlap = 18.87%, non-M1 overlap = 9.28%; Participant B M1 overlap = 1.94%, non-M1 overlap = 12.46%).

### Exploratory CART Analysis

**Figure 5:**
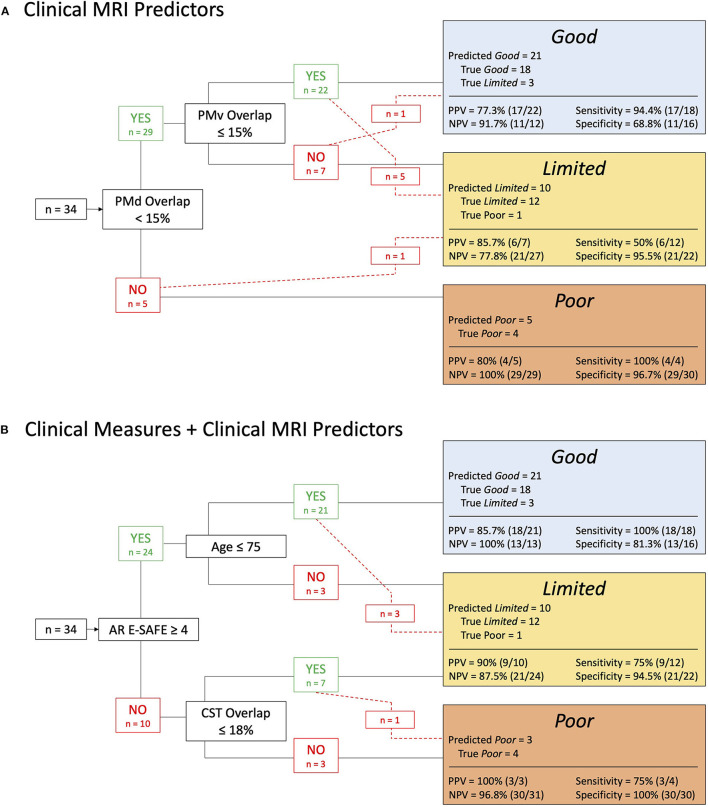
**(A)** Dorsal (PMd) and ventral (PMv) premotor tract overlap predicts paretic upper extremity (PUE) outcome category with 79% accuracy. **(B)** Estimated shoulder abduction finger extension manual muscle test score assessed at admission to acute inpatient rehabilitation (AR E-SAFE), patient age, and corticospinal tract (CST) lesion overlap % predicts PUE outcome category with 88% accuracy. PPV, positive predictive value; NPV, negative predictive value.

When only MRI-derived metrics were made available for an exploratory CART analysis, it yielded a decision tree selecting SMATT PMd tract overlap <15% and SMATT PMv tract overlap ≤ 15% to classify patients. The resulting decision tree was 79.4% accurate when decision tree predictions were tested against the outcome cluster classification (correct classification for 27 of 34 patients) (Figure 5A). SMATT PMd tract overlap <15% distinguished those in the *Poor* outcome group from *Limited* or *Good* outcome groups with 80% accuracy (4 of the 5 *Poor* PUE outcome predictions were true). The largest error was introduced when distinguishing *Limited* from *Good* outcome groups where PMv overlap ≤ 15% only did so accurately for half those in the *Limited* outcome group. Most inaccurate predictions were higher than the achieved outcome (i.e., 5 individuals predicted to be in the *Good* outcome group achieved an E-ARAT within the *Limited* outcome score range). However, 1 individual predicted to be in the *Poor* outcome group achieved an E-ARAT within the *Limited* outcome score range and 1 individual predicted to be in the *Limited* outcome group achieved an E-ARAT within the *Good* outcome score range. The resulting decision tree was 75% accurate in outcome group prediction for those with ischemic stroke (18 of 24 patients with ischemic strokes were correctly classified) and 90% accurate in outcome group prediction for those with hemorrhagic stroke (9 of 10 patients with any hemorrhagic involvement were correctly classified). See Figure 5A for further statistics on predictive values, sensitivity, and specificity.

When all clinical and MRI metrics were made available to the CART analysis as potential predictors of PUE outcome, it yielded a decision tree selecting AR E-SAFE, patient age, and SMATT CST overlap to classify patients with 88.2% accuracy (correct classification for 30 of 34 patients) (Figure 5B). For those with AR E-SAFE <4, all of whom had ischemic strokes, SMATT CST lesion overlap > 18% delineated *Poor* from *Limited* outcome groups with 90.0% accuracy (correct classification for 9 of 10 patients). However, similar error as in (A) was introduced for those with higher strength at admission to AR (AR E-SAFE > 4) where patient age > 75 years was selected to differentiate *Good* from *Limited* outcome groups but only did so accurately for half those in the *Limited* outcome group. All inaccurate predictions were higher than the achieved outcome (i.e., 3 individuals predicted to be in the *Good* outcome group achieved an E-ARAT within the *Limited* outcome score range; 1 individual predicted to be in the *Limited* outcome group achieved an E-ARAT within the *Poor* outcome score range). The resulting decision tree was 91% accurate in outcome group prediction for those with ischemic stroke (22 of 24 patients with ischemic strokes were correctly classified) and 80% accurate in outcome group prediction for those with hemorrhagic stroke (8 of 10 patients with any hemorrhagic involvement were correctly classified). See Figure 5B for further statistics on predictive values, sensitivity, and specificity.

## Discussion

Current study findings revealed that clinical MRI-derived CST lesion overlap was associated with PUE motor outcome post-stroke and that cortical projections within the CST, beyond those emanating from M1, were able to distinguish between PUE motor outcome groups. Further, results suggest that exploratory predictive models using clinical MRI metrics, either alone or in combination with clinical measures, can accurately identify recovery outcome category for patients during both the acute and early subacute phases of post-stroke recovery that underwent AR post-stroke.

### Clinically-Derived Lesion Overlap of CST Were Associated With Recovery of PUE Motor Function

Clinically-derived lesion overlap percentages for both the entire CST and specifically for the PMv CST contribution emerged as metrics with significant associations to PUE outcome at 90 days post-stroke. This observation is in agreement with previous studies employing higher-resolution MRI that showed functional PUE outcome was correlated with extent of injury to both M1 (26) and non-M1 tracts (16, 25, 26, 39, 62). However, our retrospective study provides evidence that lower-resolution, routinely available clinical scans may provide imaging-based information with prognostic utility for PUE motor outcome post-stroke. Our results indicate there may be advantages to evaluating the structural status of tracts outside of M1 CST and are in agreement with those from a prior study where CST integrity of the tract projecting to PMd was positively correlated with grip strength post stroke (39), and a recent study wherein connectivity between M1, premotor, supplementary motor and parietal areas was necessary for more robust PUE recovery post-stroke and was particularly important for those with greater motor impairment (29). Also in keeping with findings from previous studies (25, 26), lesion volume was not significantly associated with PUE outcome suggesting lesion location may be a more important factor contributing to PUE motor function than total lesion volume.

Current findings suggest that PUE functional outcome level is likely to be higher when there is a smaller extent of CST injury, in particular when PMv descending CST injury is minimal. This novel finding aligns with the role of PMv in upper limb planning and control. The PMv has been implicated in proper anticipatory shaping of the hand for grasping actions in both non-human primates and humans and several subtests of the ARAT require grasping an object to complete the task (19, 63, 64). Further, studies in non-human primates have shown that CST projections from PMv differentially terminate in upper cervical segments to potentially provide a unique contribution to control of the head, neck, and/or shoulder musculature necessary for reaching tasks (11, 19). Additionally, intracortical stimulation of the area within PMv with the densest direct connectivity to upper cervical segments elicited movement in the thumb and fingers (11, 19). Thus, stroke-related disruption of these direct PMv CST projections may underlie specific deficits resulting in poorer execution of functional reaching and grasping tasks that affect PUE recovery outcome level.

Our results underscore the relevance of contributions to the CST from cortical motor areas beyond M1, though most differences elucidated by individual contributing tracts in our study were between the highest- and lowest-functioning outcome groups. However, closer inspection of group differences in CST lesion overlap revealed that with the exception of one patient, individuals with greater than 20% disruption to PMd had a *Poor* outcome. These findings are in concert with another recent study wherein PMd lesion load was found to be the most robust neuroimaging predictor of 6-month PUE motor impairment (16). Authors from that study posited that the significant influence of PMd projections on motor recovery post-stroke may be due to the similar relative size of CST projections from M1 and PMd and their similar activation during complex motor tasks (16). Our results are further corroborated by a prior study that associated PMd lesion load with reduced grip strength post stroke (39).

PMd is commonly thought to be a motor planning center because of its known contributions to cognitive aspects such as spatial attention and working memory and its many projections to M1 (19, 20). PMd receives inputs from supplementary motor areas as well as parietal and prefrontal cortices, likely illustrating some role in integration and planning prior to motor execution (19). However, PMd also acts to control the execution of movement and contributes to descending motor signals both indirectly *via* its connections with M1 and directly through its CST projections (19, 20). Though the termination site of these direct projections are less clear in humans, animal literature has shown that direct projections from PMd to the spinal cord terminate on interneurons, descending subcortical motor networks, and in the region of motoneurons which may imply a more complex modulatory role in motor execution, one that has a major impact on motor performance (12, 19).

In a previous study (32), a single participant achieved minimal actual recovery of PUE motor function though the predicted outcome by an earlier iteration of PREP was expected to be notable (equivalent of a Good outcome in later studies). This individual displayed acute weakness (SAFE <5) but was MEP+ during TMS assessment of M1 CST functional integrity (32). The authors speculated that part of the rationale for the overestimation of PUE outcome was due to “isolated and complete” damage to the premotor cortex which would not have been detectible using M1 CST based lesion analysis nor TMS assessment targeting M1 (32). Our findings support the notion that disruption to non-M1 motor areas may influence post-stroke recovery, particularly in patients with more profound PUE impairment, and highlight the potential utility of further investigating projections within the CST beyond those from M1.

### Clinical MRI-Based Metrics of Acute Post-stroke CST Status Identified Recovery Outcome Category in Patients Undergoing AR

Clinical MRI-derived CST lesion overlap may offer an earlier indication of PUE outcome (as early as 24 h post-stroke) than previously established clinical measures of PUE strength (42). Despite previous work using clinical MRI showing that M1 CST bore the strongest association with PUE outcomes (27), here we demonstrated that lesion overlap in non-M1 CST contributors (PMd and PMv tracts) were able to distinguish PUE outcome groups and did so with similar predictive accuracy for both ischemic and hemorrhagic stroke. When considering the clinical utility of outcome predictors, accurate assessments available during acute hospitalization may be preferable for early clinical decision making to optimize resource management. In the timeframe of acute hospitalization, our findings indicated that non-M1 projections within the CST offered the strongest predictor of PUE outcome suggesting that prospective evaluation of clinical MRI-based CST metrics is warranted to determine if lesion involvement in M1 and/or non-M1 cortical projections is predictive of PUE recovery.

CST lesion overlap improved predictions using clinical metrics alone. In our cohort, PUE outcome predictions made using a combination of clinical measures and MRI biomarkers (AR E-SAFE, patient age, CST lesion load) showed improved PUE outcome prediction accuracy over use of either clinical metrics or MRI metrics alone. Our findings are in close agreement with a recent study that found that the combination of initial PUE impairment, patient age, and PMd CST structural integrity was a strong predictor of 6-month PUE impairment (16). The current findings are in line with previous finding showing: (1) PUE strength is a gross measure of baseline impairment that provides a general indication of the capacity to generate force required for functional task performance; (2) both initial impairment and patient age are predictors of functional motor outcome (3, 65–68); and (3) CST structural integrity provides insight into the underlying neural resources available for spontaneous biological recovery and experience-dependent plasticity in addition to more specific information regarding resources for sensorimotor control, motor planning, sequencing, and execution (66, 68). Quantifying CST structural integrity post-stroke may be particularly important for those with initially-lower levels of volitional control as the resolution of early strength deficits is likely to be significantly influenced by CST tract status (2, 32, 43, 44). Lesions localized within the CST are frequently associated with more severe, persistent loss of PUE motor function than lesions in other sites suggesting that certain areas of the brain and/or neuronal cell constituents may be more amenable to spontaneous biological recovery and/or plastic reorganization after stroke (7, 22–25, 27). Disruption of CST from non-M1 cortical contributors may cause a loss of unique modulatory function carried out by those descending fibers rather than a total loss of premotor cortical function, as these areas also project directly to M1. However, there is not yet a functional parcellation distinguishing contributions of descending vs. M1 projections of the PM to motor control. Therefore, structural biomarkers that quantify disruption to M1 and non-M1 CST projections may offer the specificity necessary to differentiate PUE functional recovery outcome categories however, they do not yet allow us the specificity to predict loss of specific domains of motor planning, execution or refinement. Additional studies are needed to further characterize potential tract-based biomarkers of domain-specific motor recovery.

### Limitations

Our retrospective study design has strengths and limitations. An advantage of the retrospective study design is that it allowed for critical appraisal of current standards of clinical care and recovery outcomes within the study cohort, thus our dataset may more accurately represent the true recovery experience for patients post-stroke. However, the retrospective design also required estimation of measurements including E-ARAT performance, the primary outcome measure in our study. Although estimation may introduce some measurement error to current findings, we previously showed good inter-rater reliability for the estimation approach suggesting results were not subjected to systematic bias. It was also not possible to control for differences in the content of therapy provided at each stage of post-stroke care which may limit generalizability in comparison with previous studies. However, individuals received therapy in the same rehabilitation setting and should have received a similar dosage and type of therapeutic intervention. Further, the therapy duration across the continuum of care was found to be similar across outcome groups.

The use of lower-resolution clinical MRI data processing may have introduced error during the normalization process as co-registration to a high-resolution standard template may result in imprecise alignment with neuroanatomic structures and diminished accuracy of lesion boundary localization. The template image and tract atlas (27) used for normalization in our study was derived from scans of 152 young, healthy individuals (mean age = 25 years) which may be less analogous to our cohort of individuals (mean age = 62 years) than an age-matched template due to known age-related changes (69, 70).” Lower resolution (larger voxel sizes) may lead to artificially larger lesion load values. The consequences could include overrepresenting lesion load in specific tracts. Relying on clinical scans, therefore, could result in missing subtle differences in lesion load compared to higher resolution images with small voxel sizes. Despite these limitations, we still observed tract-specific associations with clinical imaging routinely collected with standard-of-care management post-stroke highlighting the possible translational significance of the current findings (71).

We adopted a conservative threshold for statistical significance, which may have increased the likelihood of type 2 error given the size of the study cohort. Therefore, we also chose to report non-significant trends in the results. In seeking a clear MRI metric that differentiates between *Limited* and *Poor* outcomes, further prospective research with a larger cohort size may be warranted. Further, a few patients with high CST lesion overlap may have influenced the correlation between lesion load and PUE outcomes (Figure 2), however, these data points are consistent with our a priori hypotheses and corroborate previous results (16).

Lastly, although our exploratory CART analysis yielded decision trees that accurately predicted outcome for between 79 and 88% of individuals, the small sample size and category distribution may have led to overfitting of the model. Automated pruning was utilized to avoid overfitting of CART results, but predictive accuracy of decision trees created by the CART analysis were not tested using an independent testing data set, which could limit generalization of findings to other patient populations. Thus, definitive conclusions on the predictive merit of these decision trees should be viewed as a preliminary guide to future larger-scale prospective studies. CART results and predictions based upon retrospective chart review enable clinicians to make decisions that are historically consistent but may not be optimal for care planning and management. Further investigation and validation of predictive models using larger datasets will be necessary to confirm these preliminary study findings.

## Conclusions

The current findings indicate that biomarkers of CST integrity derived from routinely-available clinical MRI are associated with level of recovery of PUE function and may provide additional information to inform predictive models of functional outcome. Prospective studies are recommended to determine the utility of including clinical imaging-based biomarkers of white matter tract structural integrity in predictive models of post-stroke recovery. In an era of precision medicine, biologically-informed algorithms that accurately predict recovery outcome hold promise for improving care plan development, patient management, and optimized allocation of rehabilitation resources.

## Data Availability Statement

The raw data supporting the conclusions of this article will be made available by the authors, without undue reservation.

## Ethics Statement

The studies involving human participants were reviewed and approved by Emory University Internal Review Board. Written informed consent for participation was not required for this study in accordance with the national legislation and the institutional requirements.

## Author Contributions

MS and MB: project conception, research design, and data interpretation. MS: data acquisition, analysis, and manuscript preparation. MS, MB, NB, and SB: manuscript consultation, review, and approval. All authors contributed to the article and approved the submitted version.

## Funding

MS was supported by a Stroke Research Fellowship from the NIH StrokeNet.

## Conflict of Interest

The authors declare that the research was conducted in the absence of any commercial or financial relationships that could be construed as a potential conflict of interest.

## Publisher's Note

All claims expressed in this article are solely those of the authors and do not necessarily represent those of their affiliated organizations, or those of the publisher, the editors and the reviewers. Any product that may be evaluated in this article, or claim that may be made by its manufacturer, is not guaranteed or endorsed by the publisher.
