# Supplementary material for: Clinical Imaging-Derived Metrics of Corticospinal Tract Structural Integrity Are Associated With Post-stroke Motor Outcomes: A Retrospective Study
Source: Front Neurol. 2022 Feb 17;13:804133. doi: 10.3389/fneur.2022.804133 (PMC8893034; doi:10.3389/fneur.2022.804133)
Supplement: Supplementary file 1 [file Data_Sheet_1.docx]

**Appendix Table 1: MRI scanner type and sequence parameters per patient**

| **Patient ID** | **Scanner Type** | **Sequence Used** | **TR (ms)** | **TE (ms)** | **Flip Angle (deg)** | **Voxel Size (mm)** | **Matrix Size (pixels)** | **Slice Spacing (mm)** | **Number of Slices** |
| --- | --- | --- | --- | --- | --- | --- | --- | --- | --- |
| 1 | SIEMENS TrioTim | Ax T2 GRE | 482 | 25.7 | 20 | 0.90x0.90x5 | 216x256 | 6 | 27 |
| 2 | SIEMENS Espree | Ax DWI | 4800 | 107 | 90 | 1.61x1.61x5 | 150x150 | 6 | 27 |
| 3 | SIEMENS Espree | Ax DWI | 4800 | 107 | 90 | 1.61x1.61x5 | 150x150 | 6 | 27 |
| 4 | SIEMENS Espree | Ax DWI | 4800 | 107 | 90 | 1.61x1.61x5 | 150x150 | 6 | 27 |
| 5 | SIEMENS Avanto | Ax T2 GRE | 668 | 20 | 20 | 0.45x0.45x5 | 432x512 | 6 | 27 |
| 6 | GE MEDICAL SYSTEMS Signa HDxt | Ax DWI Asset | 10000 | 82.4 | 90 | 0.94x0.94x5 | 256x256 | 6 | 28 |
| 7 | SIEMENS TrioTim | Ax DWI | 4400 | 91 | 90 | 1.20x1.20x5 | 192x192 | 6 | 27 |
| 8 | SIEMENS Avanto | Ax DWI | 4300 | 92 | 90 | 1.60x1.60x5 | 150x150 | 6 | 28 |
| 9 | SIEMENS Espree | Ax FLAIR | 8000 | 132 | 180 | 0.47x0.47x5 | 400x512 | 6 | 27 |
| 10 | SIEMENS Avanto | Ax DWI | 4200 | 92 | 90 | 1.60x1.60x5 | 150x150 | 6 | 27 |
| 11 | SIEMENS Espree | Ax FLAIR | 8000 | 132 | 180 | 0.47x0.47x5 | 400x512 | 6 | 27 |
| 12 | SIEMENS Avanto | Ax T2 FLAIR BR | 8000 | 130 | 180 | 0.45x0.45x5 | 400x512 | 6 | 27 |
| 13 | SIEMENS TrioTim | Ax DWI | 4400 | 91 | 90 | 1.20x1.20x5 | 192x192 | 6 | 27 |
| 14 | SIEMENS Espree | Ax FLAIR | 8000 | 132 | 180 | 0.47x0.47x5 | 400x512 | 6 | 27 |
| 15 | SIEMENS Espree | Ax DWI | 5100 | 107 | 90 | 1.61x1.61x5 | 150x150 | 6 | 29 |
| 16 | SIEMENS TrioTim | Ax DWI | 4400 | 91 | 90 | 1.20x1.20x5 | 192x192 | 6 | 27 |
| 17 | SIEMENS Avanto | Ax DWI | 4000 | 92 | 90 | 1.53x1.53x5 | 150x150 | 6 | 25 |
| 18 | SIEMENS Espree | Ax T2 GRE | 552 | 25.6 | 20 | 0.75x0.75x5 | 270x320 | 6 | 27 |
| 19 | GE MEDICAL SYSTEMS Signa HDxt | Ax DWI Asset | 8000 | 81 | 90 | 1.02x1.02x5 | 256x256 | 5 | 32 |
| 20 | SIEMENS Avanto | Ax DWI | 4200 | 92 | 90 | 1.53x1.53x5 | 150x150 | 6 | 27 |
| 21 | SIEMENS TrioTim | Ax DWI | 4400 | 91 | 90 | 1.20x1.20x5 | 192x192 | 6 | 27 |
| 22 | SIEMENS Espree | Ax T2 GRE | 1130 | 25.6 | 20 | 0.84x0.84x5 | 320x290 | 6 | 29 |
| 23 | SIEMENS Avanto | Ax T2 GRE | 549 | 25.7 | 20 | 0.90x0.90x5 | 216x256 | 6 | 29 |
| 24 | SIEMENS Avanto | Ax DWI | 4200 | 92 | 90 | 1.60x1.60x5 | 150x150 | 6 | 27 |
| 25 | SIEMENS TrioTim | Ax T2 GRE BR | 668 | 20 | 20 | 0.47x0.47x5 | 432x512 | 6 | 27 |
| 26 | SIEMENS TrioTim | Ax DWI | 4400 | 91 | 90 | 1.20x1.20x5 | 192x192 | 6 | 27 |
| 27 | SIEMENS TrioTim | Ax T2 GRE | 668 | 20 | 20 | 0.45x0.45x5 | 432x512 | 6 | 27 |
| 28 | SIEMENS Avanto | Ax DWI | 4146 | 92 | 90 | 1.60x1.60x5 | 150x150 | 6 | 27 |
| 29 | SIEMENS TrioTim | Ax T2 GRE | 668 | 20 | 20 | 0.47x0.47x5 | 432x512 | 6 | 27 |
| 30 | SIEMENS Avanto | Ax T2 GRE | 516 | 25.7 | 20 | 0.90x0.90x5 | 216x256 | 6 | 29 |
| 31 | SIEMENS TrioTim | Ax T2 GRE BR | 693 | 20 | 20 | 0.47x0.47x5 | 432x512 | 6 | 28 |
| 32 | SIEMENS TrioTim | Ax DWI | 4400 | 91 | 90 | 1.20x1.20x5 | 192x192 | 6 | 27 |
| 33 | SIEMENS TrioTim | Ax DWI | 4646 | 91 | 90 | 1.30x1.30x5 | 192x192 | 6 | 29 |
| 34 | SIEMENS Espree | Ax DWI | 5100 | 107 | 90 | 1.61x1.61x5 | 150x150 | 6 | 29 |

# APPENDIX A. ARAT ESTIMATION METHODOLOGY

## A.1 Methods

Action Research Arm Test (ARAT) scores were estimated from therapy documentation at approximately 90 days post-stroke in accordance with the grading criteria for each test. Estimated ARAT (E-ARAT) scoring was conducted by two licensed, clinical neurologic therapists who were otherwise blinded to study findings. Rehabilitation provider notes were evaluated in detail to extract the following measures, where available, for each patient: clinical assessments of PUE muscle and grip strength, coordination, active and passive range of motion, observational movement analysis, therapeutic activity, exercises performed, rehabilitation goals, Nine-Hole Peg Test (9HPT) and Box and Block Test (BBT) scores as compared to matched, normative values.(1–5)

The test is composed of 4 sub-tests with 19-items in total and is conducted using the affected upper extremity; each item is scored on a 4-point ordinal, 0-3 scale.(6) A score of 3 indicates that an individual performed the task with “normal” motion, completing it within 5 seconds.(6) A score of 2 indicates an individual was able to complete the task but either 1) had difficulty with task completion, requiring compensatory motion or 2) the time to completion was longer than normal (5 - 60 seconds).(6) A score of 1 indicates only partial performance of the task within 60 seconds, and a score of 0 indicates the individual could not initiate any part of the task within 60 seconds.(6) The first item in each subtest is the most challenging and is followed by the least challenging task, enabling streamlined testing (if the individual scores a 3 on the first item, a full score for that sub-test is recorded or; if the individual scores a 0 on the first two items, the entirety of the sub-test is graded as a 0).(6) All items and sub-tests are listed in the ARAT scoring sheet (**Figure A.1**). The test is conducted with the individual seated at a desk in a neutral position.(6) Grasp subscale tests require the individual to grasp objects of varying sizes and place them on a shelf 37-cm above the desktop.(6) Grip subscale items are all performed at table-level, again with differently sized and shaped items.(6) Pinch subscale tasks require the individual to pinch either a ball-bearing or marble, lifting it from the top of the shelf to place it on the desktop; tests are conducted using specified digits testing thumb opposition motion with index, middle, and ring fingers.(6)

Figure A.1 – ARAT Scoring Sheet. From Yozbatiran et. al., 2008.

As scores for each item were estimated, all medical records taken at and around day-90 post-stroke were utilized to aid in estimation. Each ARAT estimator independently reviewed the electronic medical record (EMR) and determined maximal and minimal scores for each ARAT test item, creating a score range for every patient. E-ARAT for every patient was calculated by taking each estimator’s median score and averaging the two values. Scoring for two patients from our cohort are described below.

### A.1.1 ARAT Estimation - Patient Example 1

The occupational therapy treatment note states that the patient demonstrates full active range of motion (AROM) of the affected upper extremity. The patient performs the 9HPT in 25 seconds (normative value for the patient’s sex and age is 22.29 seconds).(1) Patient’s grip strength measured via hand-held dynamometer is 66.3 lbs, and the normative value for hand, sex, and age is 76.8 lbs.(5) The patient performed tasks including “sustained overhead activity placing nuts and bolts together on board on middle shelf,” “1# wrist weight to L UE to complete overhead placement of 1” cubes on shelf,” and “fine motor coordination task placing clothespins and pennies in ruler with minimal difficulty.” The occupational therapist documented assessments such as, “patient making good progress towards goals. Continues to have mild decrease in LUE strength and coordination,” and “patient with good participation, fatigue noted with increased weight, but good form.”

Considering the therapist-chosen treatment interventions and demonstration of full AROM on assessment, the member of the research team estimating the ARAT score could reasonably assume the patient possessed sufficient anti-gravity shoulder strength and range of motion to perform items on the gross movement subscale within 5 seconds and without compensation, scoring 9/9 points. The patient’s participation in advanced activities including overhead reaching, object manipulation, and grasping indicate they would likely score 3/3 on some of the grasping and griping subtests. The patient does have decreased grip strength when compared to age- and sex-matched norms, however, grip strength of 66.3 lbs would be sufficient to complete grip and grasp tasks without difficulty. The occupational therapist does note coordination deficits throughout interventions and outcome assessments, suggesting the patient may use minor compensatory movements or require increased time to complete the initial, more challenging items. As a result, the patient was scored within a minimum of 15/18 and a maximum of 18/18 on the grasp subscale and a similar 10-12/12 range on the grip subscale. Finally, the patient was engaged in tasks involving pinching and manipulating small objects with “minimal difficulty” and performed the 9HPT marginally slower than normative values. Therefore, the patient would likely have scored a 2/3 on the more challenging pinch subtests but 3/3 on the less challenging items, resulting 15-18/18 range on the pinch subscale. Summing the minimum and maximum potential scores yields an E-ARAT score range of 49-57/57. The median of this range is 53 which would be the E-ARAT score attributed to this patient for one of the examiners. The same scoring was independently conducted by a second examiner; 53 would then be averaged with the median score of the second examiner to yield the E-ARAT score for this patient.

### A.1.2 ARAT Estimation - Patient Example 2

For another patient in our cohort, the occupational therapist set goals primarily related to proper positioning of the impaired arm, increasing grip strength to 10 pounds, increasing active shoulder flexion 10 degrees, the ability to manipulate 12 blocks on the Box and Blocks Test,(2) and using the unimpaired arm in techniques to care for and facilitate function in the impaired arm. Therapeutic exercises and activities were performed in gravity-minimized positions with therapist or patient self-assistance using the unimpaired arm. One treatment session was focused on “bringing object to [the] mouth and then reaching forward with R UE.” The grip strength goal was not met by 90-days post-stroke (patient achieved a maximum of 7 pounds of grip strength). The patient did achieve 10 degrees of active shoulder flexion but was not able to complete the Box and Blocks Test.(2) The occupational therapist notes that the “hand continues to be extremely limited by decreased shoulder strength.”

Using this information, the member of the research team estimating the ARAT score could assume that the patient had sufficient isolated shoulder and finger muscle activation to initiate all of the items on each subscale, thus achieving a minimal score of 1/3 on each item. The patient was also able to perform the “hand to mouth” item of the gross movement scale, as this was specifically indicated within one of the treatment interventions. Given the gross shoulder weakness however, it is likely the patient would rely on extensive compensation to perform any actions within the gross movement subscale, resulting in a score range 3-5/9. As the patient demonstrates considerable proximal weakness with some preservation of hand and finger dexterity, it is possible they would perform best on the grip subscale, as it does not require movement of the object from desk-height to shelf-height, as the grasp and pinch subscales do. Though the patient would not be able to perform any of the grip subscale tasks within the 5-second requirement and without motor compensation, it is possible the patient would achieve a 2/3 on the easier items, thus completing the task with increased time and/or some compensatory motion. This would result in a score range of 5-7/12 for the grip subscale. As stated previously, the grasp and pinch subscales both involve moving the object from the desk-to-shelf or shelf-to-desk, requiring anti-gravity shoulder strength this patient was not documented to have. Therefore, the patient would be likely to score a 1/3 for initiation of many of the grasp and pinch subscales while successfully completing a few of the easier subtests (2/3). This would result in a score range of 6-10/18 on the grasp and a 6-8/18 on the pinch subscales, for a total score range of 18-30/57. The median of this range is 24 which would then be averaged with the other ARAT examiner to yield the E-ARAT score for this patient.

**References**

1. Oxford Grice K, Vogel KA, Le V, Mitchell A, Muniz S, Vollmer MA. Adult norms for a commercially available Nine Hole Peg Test for finger dexterity. *Am J Occup Ther* (2003) **57**:570–573. doi: 10.5014/ajot.57.5.570

2. Mathiowetz V, Volland G, Kashman N, Weber K. Adult norms for the Box and Block Test of manual dexterity. *Am J Occup Ther* (1985) **39**:386–391. doi: 10.5014/ajot.39.6.386

3. Ekstrand E, Lexell J, Brogårdh C. Test-Retest Reliability and Convergent Validity of Three Manual Dexterity Measures in Persons With Chronic Stroke. *PM R* (2016) **8**:935–943. doi: 10.1016/j.pmrj.2016.02.014

4. Lin KC, Chuang LL, Wu CY, Hsieh YW, Chang WY. Responsiveness and validity of three dexterous function measures in stroke rehabilitation. *Journal of Rehabilitation Research and Development* (2010) doi: 10.1682/JRRD.2009.09.0155

5. Crosby CA, Wehbé MA. Hand strength: Normative values. *The Journal of Hand Surgery* (1994) **19**:665–670. doi: 10.1016/0363-5023(94)90280-1

6. Yozbatiran N, Der-Yeghiaian L, Cramer SC. A standardized approach to performing the action research arm test. *Neurorehabilitation and Neural Repair* (2008) doi: 10.1177/1545968307305353
